# Duck HERC3 Positively Regulates RIG-I/MAVS-Mediated IFN-β Production Through Interaction with MAVS

**DOI:** 10.3390/ani16213329

**Published:** 2026-10-22

**Authors:** Guoyao Wang, Yujiao Guo, Jinlu Liu, Xuming Hu, Qi Xu

**Affiliations:** 1 College of Animal Science and Technology, Yangzhou University, Yangzhou 225009, China; 2 Joint International Research Laboratory of Agriculture and Agri-Product Safety, The Ministry of Education of China, Yangzhou University, Yangzhou 225009, China; 3 Key Laboratory for Evaluation and Utilization of Poultry Genetic Resources of Ministry of Agriculture and Rural Affairs, Yangzhou University, Yangzhou 225009, China

**Keywords:** HERC3, MAVS, IFN-β, duck, innate immunity

## Abstract

The RIG-I-like receptor (RLR) pathway is essential for detecting cytosolic viral RNA and initiating type I interferon responses via the key adaptor MAVS. Although MAVS regulation has been extensively studied in mammals, little is known about its regulatory mechanisms in avian species. HERC3 participates in the regulation of RLR-mediated immunity in mammals, but its role in ducks remains unexplored. Here, we investigated the function of duck *HERC3* (*duHERC3*) in the RLR pathway. We found that *duHERC3* was ubiquitously expressed in all examined tissues and significantly upregulated upon Newcastle disease virus (NDV) infection and 3p-hpRNA stimulation. Overexpression of duHERC3 elevated the mRNA levels of MAVS and OASL and enhanced IFN-β production, whereas knockdown of *duHERC3* exerted opposite effects. Co-immunoprecipitation (Co-IP) revealed that duHERC3 interacts with the N-terminal domain of MAVS (aa5-175). Moreover, duHERC3 enhanced MAVS-mediated IFN-β induction in a RIG-I-dependent manner, indicating that duHERC3 functions through the canonical RIG-I-MAVS axis. Collectively, our findings demonstrate that duck HERC3 positively regulates MAVS-dependent IFN-β production via interaction with MAVS, providing new insights into the regulatory network of avian innate immunity.

## Simple Summary

Ducks serve as important natural reservoirs for multiple viruses, including influenza viruses. They can rapidly recognize viral infections via the innate immune system and initiate antiviral defense responses such as interferon production. Understanding how ducks resist viral infection reveals that proteins including RIG-I and MAVS form a critical signal transduction pathway, yet the fine-tuned regulation of this pathway in ducks remains largely uncharacterized. In this study, we identified duck HERC3 as a protein that potentiates antiviral immune responses. HERC3 is expressed in all duck tissues examined, and its expression is markedly upregulated following infection with Newcastle disease virus or treatment with viral mimetic stimuli. Elevation of HERC3 levels in cells increases MAVS expression and promotes IFN-β production, whereas HERC3 knockdown yields the opposite effects. We further demonstrated that HERC3 interacts with MAVS, a key immune signaling protein. Moreover, duHERC3 enhances MAVS-mediated IFN-β induction in a RIG-I-dependent manner. These findings identify HERC3 as a novel positive regulator of the duck antiviral immune response, advance our understanding of avian immunity, and provide new insights for investigating avian antiviral defense mechanisms and developing strategies for related disease prevention and control.

## 1. Introduction

Pattern recognition receptors (PRRs), which are critical to innate immunity as the first line of defense against viruses, are responsible for the initiation of the antiviral response by the rapid recognition of molecular patterns present in viruses during infection [1]. Among cytosolic PRRs, retinoic acid-inducible gene I (RIG-I)-like receptors (RLRs), encompassing 3 members—RIG-I, melanoma differentiation-associated protein 5 (MDA5) and laboratory of genetics and physiology 2 (LGP2)—are key sensors of viral infection [2]. RIG-I is a cytosolic innate immune receptor that plays a central role in the rapid response to infections by RNA viruses [3]. The binding of RNA viral ligands causes RIG-I to extend its N-terminal caspase activation and recruitment domains (CARDs) into solution, where they are then free to interact with homologous CARDs on the mitochondrial antiviral signaling protein (MAVS) [4]. The interaction between RIG-I and MAVS is thought to initiate the assembly of a MAVS oligomeric complex [5] that stimulates translocation of transcription factors IRF3 and NF-κB [6] into the nucleus, thereby producing type I interferons and ISGs [7] and performing antiviral defense [8,9].

HERC3 belongs to a subfamily of four small HERC proteins, HERC3 to 6. It contains a single amino-terminal RCC1-like domain (RLD) and a carboxyl-terminal HECT domain [10]. Recent studies found that HERC3 promotes degradation of c-Myc modulator MM1, thus inhibiting cell senescence [11]. In mammals, HERC3 facilitates RelA protein degradation, establishing HERC3 as a candidate regulator of NF-κB-mediated inflammatory responses [12]. Nevertheless, it remains unknown whether HERC3 modulates antiviral innate immunity via its interaction with MAVS, a central component of the RLR signaling cascade, especially in avian species.

Ducks are natural hosts and reservoirs of influenza viruses, which typically cause asymptomatic infection and little pathology. Although most influenza strains can replicate in mallards, they typically do not cause substantial disease in this host [13]. Notably, mallards are generally resistant to disease: their early and robust innate immune response effectively restricts viral replication and clears highly pathogenic avian influenza viruses, whereas the same strains can cause severe systemic infections and even death in humans, chickens, and other duck species. Rapid initiation of innate immune responses depends on viral recognition by pattern recognition receptors (PRRs) expressed in tissues where the virus replicates. RIG-I, which detects RNA ligands derived from uncapped viral transcripts and initiates the IFN response, is basally expressed in a tissue-specific manner in ducks. In contrast, chickens appear to be missing RIG-I and instead detect IAV through the related RLR, MDA5 [14,15]. Interestingly, chicken cells transfected with duck RIG-I produce more IFN-β, augment expression of numerous ISGs, and restrict influenza virus [16]. Given the pivotal role of RIG-I in duck antiviral immunity, our group has been dedicated to dissecting its regulatory networks. Previous studies by the wider research community have demonstrated that the duck RIG-I-MAVS pathway is subject to modulation by multiple regulatory factors: duck TRIM25 is significantly induced upon 5′ppp dsRNA stimulation and participates in RIG-I-mediated innate immune activation [17]; duck TRIM29 interacts with duck MAVS through its C-terminal domain and inhibits IFN-β and IRF7 promoter activities in a dose-dependent manner [18]; duck PHB1 and PHB2 positively regulate IFN-β production by targeting MAVS [19]. Collectively, these findings indicate that ducks have developed multilayered regulatory networks to modulate the RLR pathway, thereby maintaining immune homeostasis during viral infection. However, whether duck HERC3 regulates the RLR pathway, particularly through MAVS interaction, remains unknown.

To explore the regulatory role of duck HERC3 in the RIG-I-MAVS antiviral signaling pathway, we first examined its expression profiles in various duck tissues upon viral infection and in duck embryo fibroblasts (DEFs) stimulated with the viral mimic 3p-hpRNA. We then evaluated the effects of HERC3 overexpression and knockdown on MAVS-mediated IFN-β production using RT-qPCR and ELISA, and identified the specific domain of MAVS that mediates its interaction with HERC3. Our findings provide new insights into the molecular regulation of avian innate immunity and expand the current understanding of the RLR signaling network.

## 2. Materials and Methods

### 2.1. Ethics Statement

All animal experiments performed in this study were approved by the Animal Ethics Committee of Yangzhou University.

### 2.2. Animals and Virus

Ten 20-day-old Cherry Valley ducks, obtained from Jiangsu Yike Food Group Co., Ltd. (Suqian, China), were randomly divided into two groups of 5 ducks each. One group was challenged with NDV via the intraorbital route at a dose of 100 μL containing 10^5^ EID_50_ NDV per duck, while the other group received an equivalent volume of phosphate-buffered saline (PBS) using the same method. At 48 h post-challenge, all ducks were anesthetized by intraperitoneal injection of sodium pentobarbital and subsequently euthanized by exsanguination. Immediately, tissue samples were collected and stored at −80 °C, while blood samples were collected in anticoagulant tubes and stored at −20 °C. The Newcastle disease virus (NDV) strain used in this study is the standard virulent strain F48E8.

### 2.3. Cell Culture

Duck embryonic fibroblasts (DEFs; ATCC, Manassas, VA, USA), DEF stably expressing EGFP and DEF stably expressing RIG-I (DEF^EGFP^ and DEF^RIG-I^, preserved in our laboratory), were cultured in Eagle’s Minimum Essential Medium (EMEM; ATCC, Manassas, VA, USA) supplemented with 10% fetal bovine serum (FBS; Gibco, Grand Island, NY, USA) and 1% penicillin-streptomycin (NCM Biotech, Suzhou, China). All cells were cultured in a humidified cell incubator at 37 °C with 5% CO_2_. All cells were confirmed to be free of mycoplasma contamination by testing.

### 2.4. Plasmid Construction and RNA Interference

The full-length coding sequence of *HERC3* was cloned into the pcDNA3.1-HA vector, while truncated MAVS mutants were generated in the pcDNA3.1-FLAG vector. The primers utilized for constructing all eukaryotic expression plasmids are listed in Table 1. The pcDNA3.1-MAVS-FLAG plasmid was maintained in our laboratory. In total, 4 siRNAs targeting *duHERC3*, together with a negative control siRNA (siNC), were chemically synthesized by GenePharma (Suzhou, China), with sequences provided in Table 2. All plasmid transfections and siRNA transfections were performed using Lipofectamine 3000 (Invitrogen, Carlsbad, CA, USA).

### 2.5. RNA Extraction and Real-Time Quantitative PCR (RT-qPCR)

The total RNA was extracted from harvested cells using RNA-isolating reagent (TIANGEN, Beijing, China) according to the manufacturer’s instructions. Qualified RNA was reverse-transcribed into complementary DNA (cDNA) using HiScript II One Step RT-PCR Kit (Vazyme, Nanjing, China), and cDNA was subsequently used in real-time quantitative PCR with ChamQ Blue Universal SYBR qPCR Master Mix (Vazyme, Nanjing, China). Each amplification reaction was prepared in a total reaction volume of 20 μL, containing cDNA template, forward-reverse primer pairs, and SYBR qPCR Master Mix. Primer sequences for target genes and the reference gene were provided in Table 1. All samples were run with 3 biological replicates, alongside 3 technical replicates for each biological sample to reduce pipetting bias. Relative transcript abundance was normalized against the reference gene and calculated by the comparative 2^−ΔΔCt^ method.

### 2.6. Enzyme-Linked Immunosorbent Assay (ELISA)

Duck IFN-β protein levels in cell culture supernatants were quantified using a commercial duck IFN-β ELISA kit (YJ373650, mlbio, Shanghai, China) according to the manufacturer’s instructions. Briefly, cell culture supernatants were harvested and centrifuged at 12,000 rpm for 10 min at 4 °C to remove cell debris, and the clarified supernatants were collected for detection. All kit reagents were equilibrated to room temperature before use. Serially diluted standards and processed supernatants were added to corresponding wells, followed by incubation at 37 °C for 30 min. After washing 5 times with wash buffer, HRP-conjugated reagent was added to each well (excluding blank wells) and incubated at 37 °C for another 30 min. The plates were washed 5 times again, and chromogen solutions A and B were added for color development at 37 °C in the dark for 10 min. The reaction was terminated by adding stop solution, and the optical density (OD) was measured at 450 nm using the blank wells as zero reference. Each biological sample was assayed in 3 technical replicates. The concentration of IFN-β was calculated based on the standard curve.

### 2.7. Co-Immunoprecipitation and Immunoblot Analysis

HERC3-HA, MAVS-FLAG, and its truncation mutant constructs were co-transfected into DEF^RIG-I^ cells. At 24 h post-transfection, cells were lysed in IP lysis buffer (Thermo Fisher Scientific, Waltham, MA, USA) supplemented with protease and phosphatase inhibitor cocktails on ice for 30 min. The lysates were centrifuged at 12,000 rpm for 15 min at 4 °C, and the supernatants were collected. A 10 μL aliquot of each supernatant was reserved as the input control. The remaining lysates were divided into two equal portions and incubated with anti-HA or anti-IgG magnetic beads (Beyotime, Shanghai, China), respectively, with gentle rotation overnight at 4 °C. After 5 washes, the bound proteins were eluted by boiling in 5× SDS loading buffer. The proteins from input, IP, and IgG fractions were separated by SDS-PAGE and transferred onto PVDF membranes. Subsequently, the membranes were blocked with 5% non-fat milk for 1 h and then incubated with primary antibodies overnight at 4 °C, followed by incubation with HRP-conjugated secondary antibodies. The antibodies used in this study included: anti-FLAG (ABclonal, Cat. No.A13526, Wuhan, China), anti-HA (ABclonal, Cat. No.AE105, Wuhan, China).

### 2.8. Statistical Analysis

All data are expressed as mean ± standard deviation (SD). Statistical analyses were performed using SPSS 27.0. For multi-group comparisons within a single cell line, one-way analysis of variance (ANOVA) followed by Tukey’s post hoc test was applied. An unpaired Student’s *t*-test was used for pairwise comparisons between two groups within the same tissue or cell line. GraphPad Prism 7 was used for graph plotting. * *p* < 0.05 was considered statistically significant, ** *p* < 0.01 was considered highly statistically significant.

## 3. Results

### 3.1. Viral Infections Induce Upregulation of Duck HERC3 Expression

**Figure 1:**
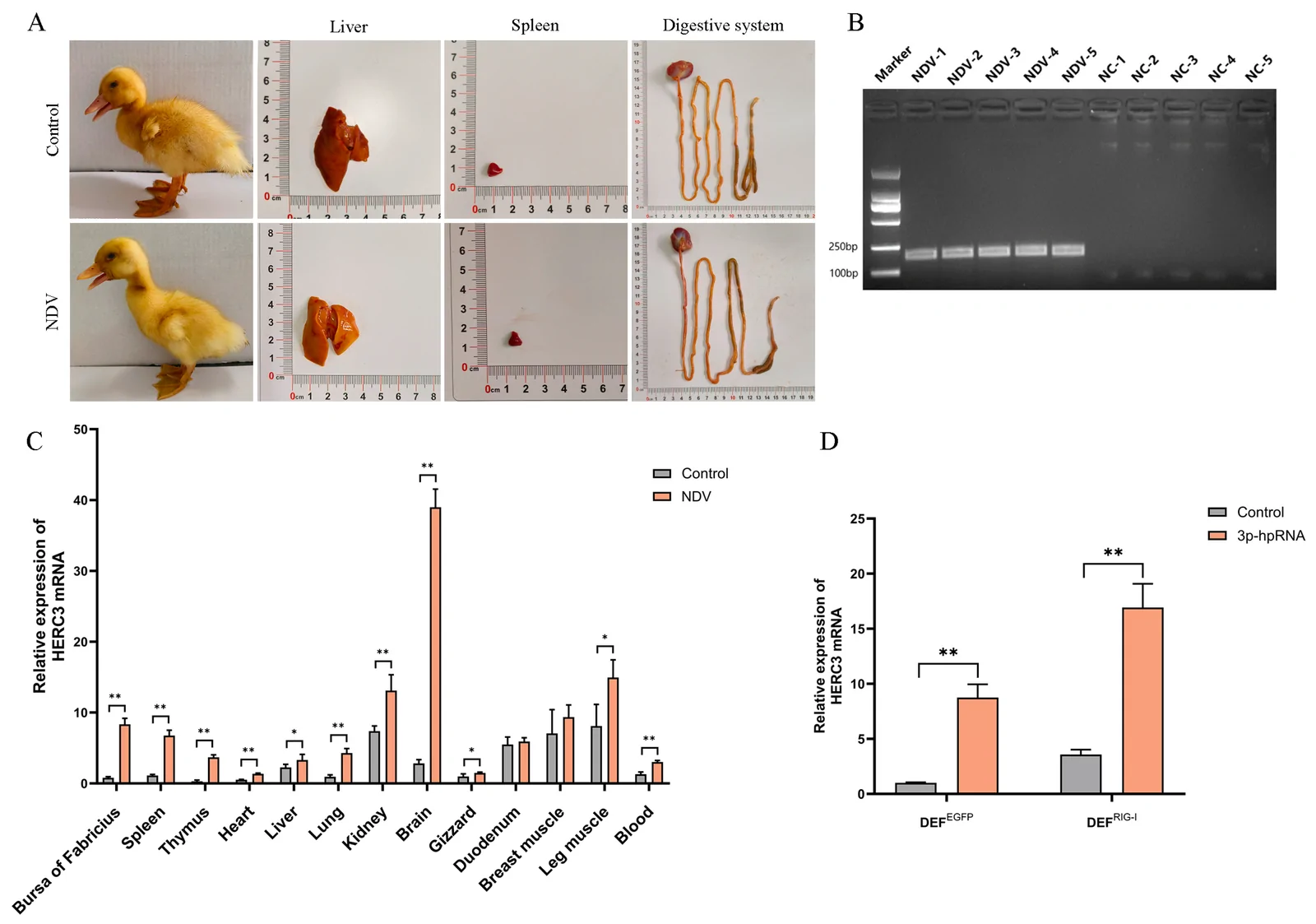
Viral infection induces upregulation of duck HERC3 expression. (**A**) Clinical signs in ducklings infected with Newcastle disease virus (NDV). (**B**) Oropharyngeal swabs were collected from ducklings prior to anesthesia, and the results were visualized by agarose gel electrophoresis; the NDV-specific amplicon is 179 bp in length. NDV: inoculated with Newcastle disease virus; NC: inoculated with PBS. (**C**) Expression levels of *duHERC3* were analyzed in tissues from PBS-inoculated (NC) and NDV-inoculated groups, including blood, bursa of Fabricius, spleen, thymus, heart, liver, lung, kidney, brain, gizzard, duodenum, breast muscle, and leg muscle. *duβ-actin* was used as the internal reference gene. Data are presented as mean ± SD (*n* = 5 ducks per group). Statistical analysis was performed using unpaired Student’s *t*-test for pairwise comparisons between NC and NDV groups within each tissue. (**D**) DEF^EGFP^: DEF cells stably expressing EGFP vector; DEF^RIG-I^: DEF cells stably expressing duck RIG-I. Cells were treated with 3p-hpRNA, an agonist of the pattern recognition receptor retinoic acid-inducible gene-I (RIG-I), for 12 h, and *duHERC3* mRNA expression levels were assessed. Data are shown as mean ± standard deviation (SD) from 3 independent experiments. Unpaired Student’s *t*-test was used for pairwise comparisons within each cell line. * *p* < 0.05; ** *p* < 0.01.

Following NDV infection, the ducks exhibited no obvious clinical signs or mortality. No apparent depression, inappetence, or neurological abnormalities were observed. However, gross examination at necropsy revealed hemorrhagic lesions in the liver (Figure 1A). We detected viral nucleic acid fragments in ducks using polymerase chain reaction (PCR). The results showed that specific bands were present in all infected groups, while no target amplification products were detected in the control group (Figure 1B). Then, we detected the expression levels of *duHERC3* mRNA in different tissues and found that *duHERC3* was ubiquitously expressed across all tissues examined. Compared with the control group, *duHERC3* expression was significantly upregulated in the bursa of Fabricius, spleen, thymus, heart, lung, kidney, brain, leg muscle, liver, gizzard, and blood of NDV-infected ducklings, while no significant changes were observed in the duodenum or breast muscle (Figure 1C). In addition, we examined *duHERC3* expression in both DEF^EGFP^ and DEF^RIG-I^ cells after stimulation with 3p-hpRNA. *duHERC3* expression was significantly elevated in both cell lines following stimulation with 3p-hpRNA (Figure 1D).

### 3.2. duHERC3 Overexpression Increases MAVS mRNA and IFN-β Production

**Figure 2:**
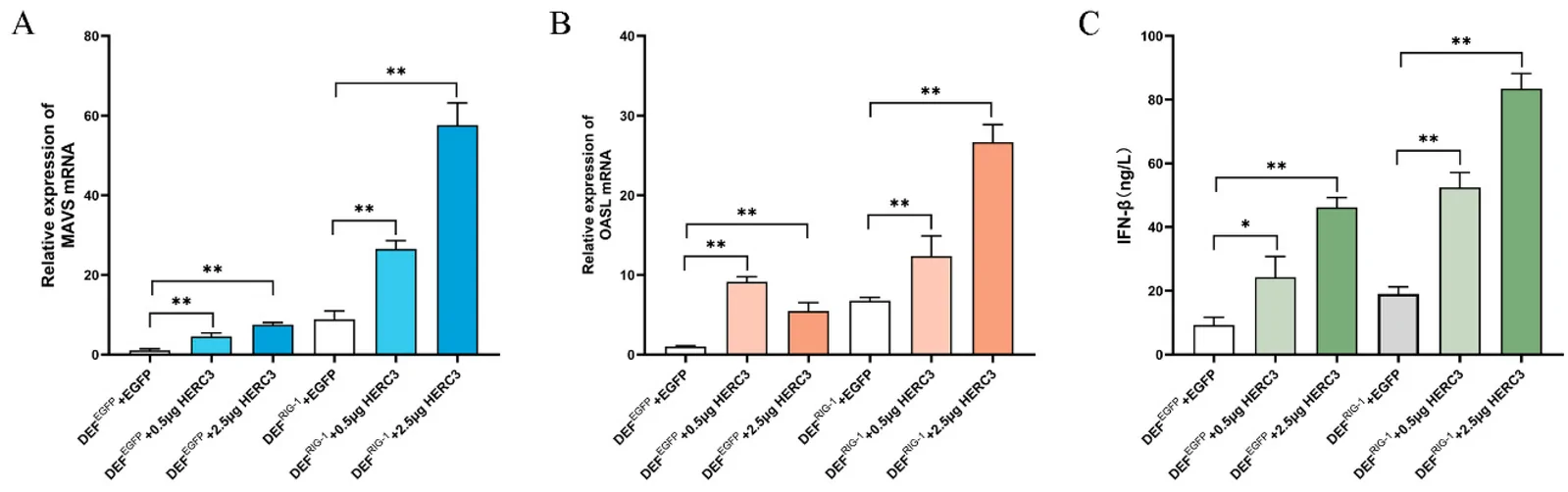
duHERC3 overexpression increases *MAVS* mRNA and IFN-β production. (**A**) MAVS expression at 24 h post-transfection. (**B**) OASL expression level at 24 h post-transfection. (**C**) IFN-β concentration in culture supernatants at 24 h post-transfection, measured by ELISA. All data are presented as mean ± SD from 3 independent experiments. Statistical analysis was performed using one-way ANOVA followed by Tukey’s post hoc test. *, *p* < 0.05; **, *p* < 0.01.

To investigate the role of duHERC3 in the duck RLR signaling pathway and its regulatory effect on IFN-β induction, we overexpressed *duHERC3* in DEF^EGFP^ and DEF^RIG-I^ cells. At 24 h post-transfection, we collected the cells and determined the mRNA levels of the interferon-stimulated gene *OASL*. Meanwhile, cell culture supernatants were harvested for IFN-β measurement via ELISA. The results showed that duHERC3 dose-dependently increased *MAVS* expression, concomitant with a significant increase in *OASL* expression. Consistently, ELISA data revealed that duHERC3 dose-dependently promoted IFN-β production. Furthermore, we found that RIG-I potentiated the duHERC3-induced expression of *MAVS*, *OASL*, and IFN-β (Figure 2).

### 3.3. Knockdown of duHERC3 Suppresses MAVS mRNA and IFN-β Production

**Figure 3:**
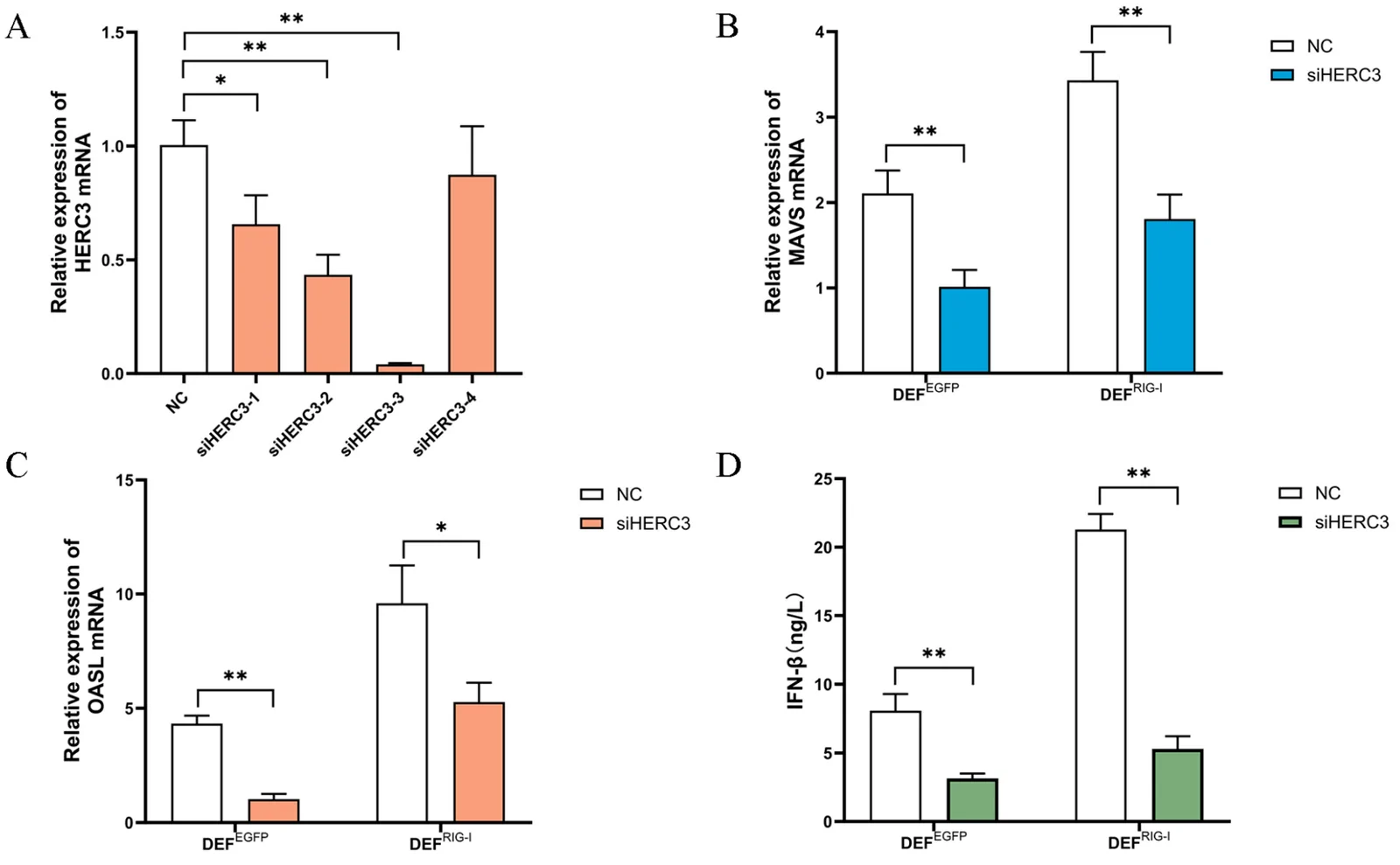
Knockdown of duHERC3 Suppresses MAVS mRNA and IFN-β Production. (**A**) Knockdown efficiency assessed by RT-qPCR at 48 h post-transfection. (**B**) *MAVS* expression level at 48 h. (**C**) *OASL* expression level at 48 h. (**D**) IFN-β concentration in supernatants at 48 h. All data are presented as mean ± SD from 3 independent experiments. Statistical analysis was performed using one-way ANOVA followed by Tukey’s post hoc test. *, *p* < 0.05; **, *p* < 0.01.

To evaluate the knockdown efficiency of duHERC3, we designed 4 siRNAs and transfected them into DEF^RIG-I^ cells and verified their interference effects. The results showed that *siduHERC3-3* exhibited the highest knockdown efficiency and was therefore selected for subsequent experiments (Figure 3A). Then, *siduHERC3-3* was transfected into DEF^EGFP^ and DEF^RIG-I^ cells, and cells and supernatants were collected at 48 h post-transfection. RT-qPCR and ELISA analyses revealed that knockdown of *duHERC3* suppressed the expression of *MAVS* and *OASL*, while also reducing IFN-β production (Figure 3B–D).

### 3.4. duHERC3 Interacts with the N-Terminus of MAVS

**Figure 4:**
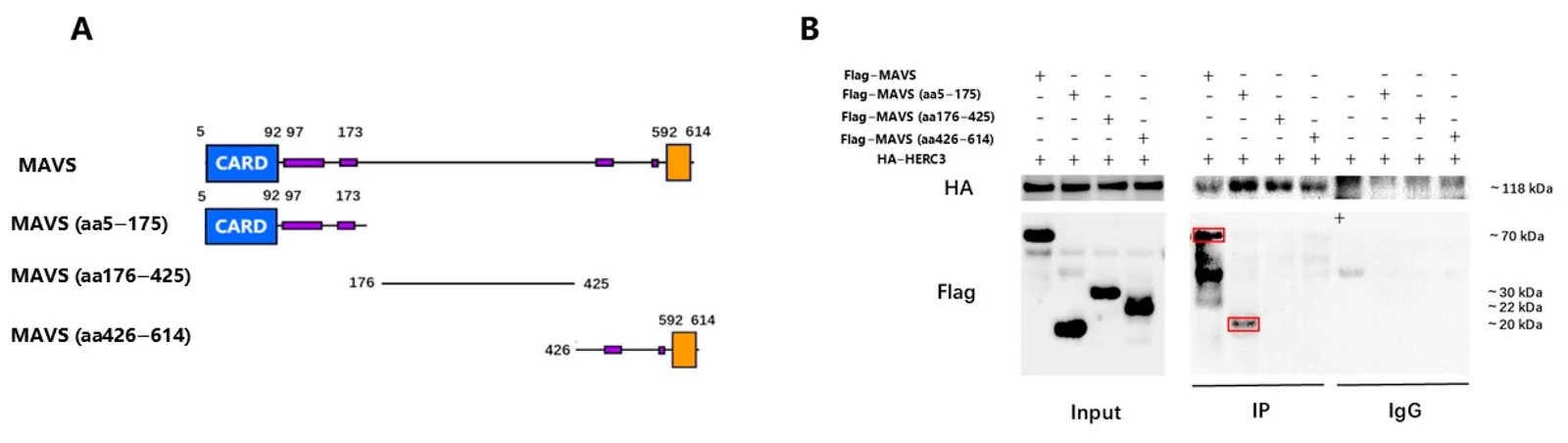
duHERC3 interacts with the N-Terminus of MAVS. (**A**) Schematic representation of duck MAVS domain architecture. (**B**) DEF^RIG-I^ cells were co-transfected with duHERC3-HA and full-length or truncated FLAG-tagged MAVS constructs. At 24 h post-transfection, cell lysates were incubated with anti-HA magnetic beads, and co-precipitated proteins were detected by Western blotting.

To investigate the interaction between duHERC3 and full-length MAVS and its 3 domains, duHERC3-HA and MAVS-FLAG and its truncated mutants were co-transfected into DEF^RIG-I^ cells (Figure 4A). Subsequently, co-immunoprecipitation (Co-IP) assays were performed to detect proteins interacting with duHERC3. The cell lysates were incubated with HA magnetic beads, and MAVS and its truncation mutants were detected in the immunoprecipitated complexes by Western blotting using an anti-FLAG antibody. The results showed that MAVS and MAVS (aa5-175) were readily detected in the immunoprecipitated complexes (Figure 4B). Therefore, our findings indicate that duHERC3 interacts with the N-terminal domain of MAVS.

### 3.5. duHERC3 Enhances MAVS-Dependent IFN-β Production

**Figure 5:**
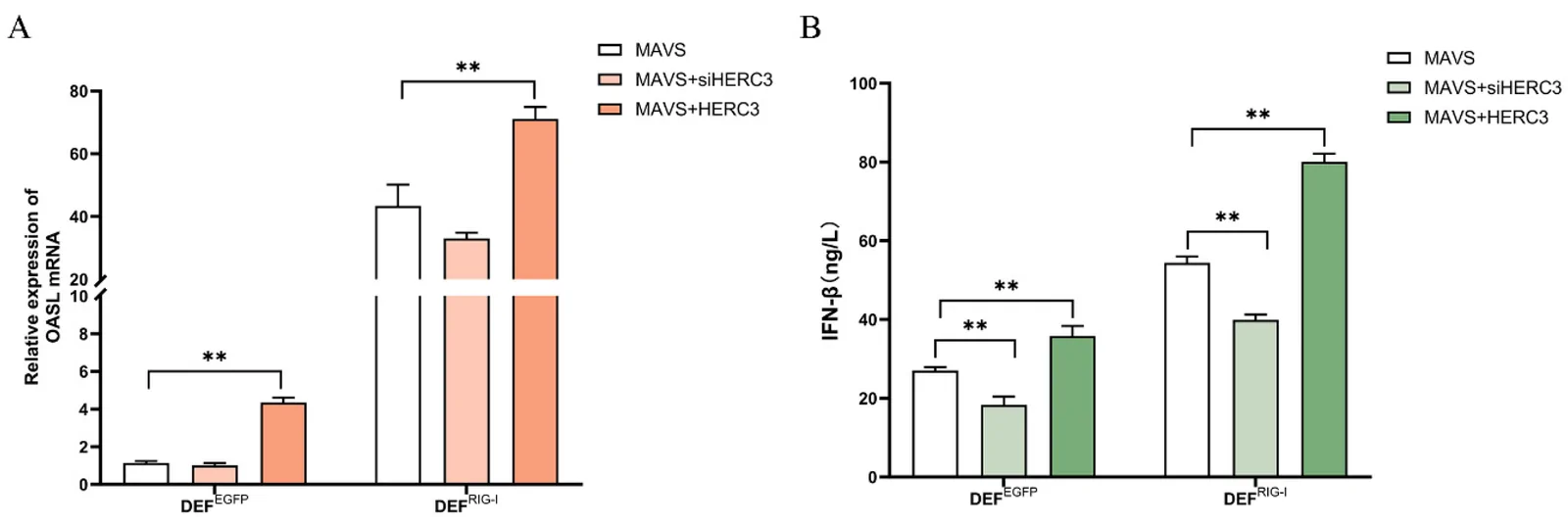
duHERC3 enhances MAVS-dependent IFN-β production. (**A**) *OASL* expression level at 48 h. (**B**) IFN-β concentration in supernatants at 48 h, measured by ELISA. All data are presented as mean ± SD from 3 independent experiments. Statistical analysis was performed using one-way ANOVA followed by Tukey’s post hoc test. ** *p* < 0.01.

To further investigate whether duHERC3 is involved in MAVS-mediated IFN-β induction, we performed the 3 treatments in DEF^EGFP^ and DEF^RIG-I^ cells (*MAVS* overexpression alone, *MAVS* overexpression combined with *siduHERC3*, and *MAVS* overexpression combined with *duHERC3*). Compared with *MAVS* overexpression alone, knockdown of *duHERC3* in the presence of *MAVS* overexpression resulted in no significant change in *OASL* expression, but significantly reduced IFN-β production. However, *MAVS* overexpression combined with *duHERC3* led to significantly elevated expression of both *OASL* and IFN-β (Figure 5). These results indicate that duHERC3 positively modulates the MAVS-dependent antiviral signaling to promote IFN-β production.

## 4. Discussion

The innate immune system serves as the first line of defense against viruses, in which the RIG-I-like receptor (RLR) signaling pathway plays a central role in recognizing cytosolic viral RNA, activating downstream cascades, and inducing the production of antiviral immune molecules [20]. In this pathway, MAVS is a critical adaptor molecule of the innate immune receptor RIG-I, bridging viral RNA recognition with antiviral signaling [21]. Although the regulatory mechanisms of MAVS activity have been extensively studied in mammals, how MAVS-mediated signaling is regulated in avian species remains poorly understood. The present study identifies duHERC3 as a positive regulator of the RLR pathway, providing a new theoretical basis for elucidating the molecular regulatory mechanisms of duck antiviral innate immunity.

Firstly, we investigated the mRNA expression levels of *duHERC3* in healthy ducks and in ducks infected with Newcastle disease virus (NDV). The results showed that *duHERC3* was ubiquitously expressed in all examined tissues. Interestingly, NDV infection significantly upregulated *duHERC3* mRNA levels in nearly all tested tissues, with particularly pronounced increases in brain and immune organs (bursa of Fabricius, spleen, and thymus). However, no significant change was observed in the duodenum or breast muscle. It has been reported that positive regulators of the RIG-I-MAVS signaling axis, SRC and PHB1/PHB2 [19,22], are also significantly upregulated in immune organs of ducks following NDV infection. In vitro, stimulation with the RIG-I agonist 3p-hpRNA significantly elevated *duHERC3* expression in both DEF^EGFP^ and DEF^RIG-I^ cells. This finding is consistent with our in vivo observation that duHERC3 is broadly upregulated in multiple tissues upon NDV infection, suggesting that duHERC3 induction may not be specific to NDV infection but rather represents a host cellular response to viral RNA-triggered innate immune signals. Notably, previous studies have demonstrated that HERC3 possesses antiviral activity at the cellular level, as HERC3 overexpression can suppress HIV-1 particle production [23]. Although duHERC3 expression was assessed at only one time point in each model (48 h post-challenge in vivo and 12 h post-stimulation in vitro), the dynamic regulation of duHERC3 has not been fully resolved. Together with the in vivo and in vitro expression changes in duHERC3 observed here, these findings suggest that duHERC3 is involved in innate immune responses.

**Figure 6:**
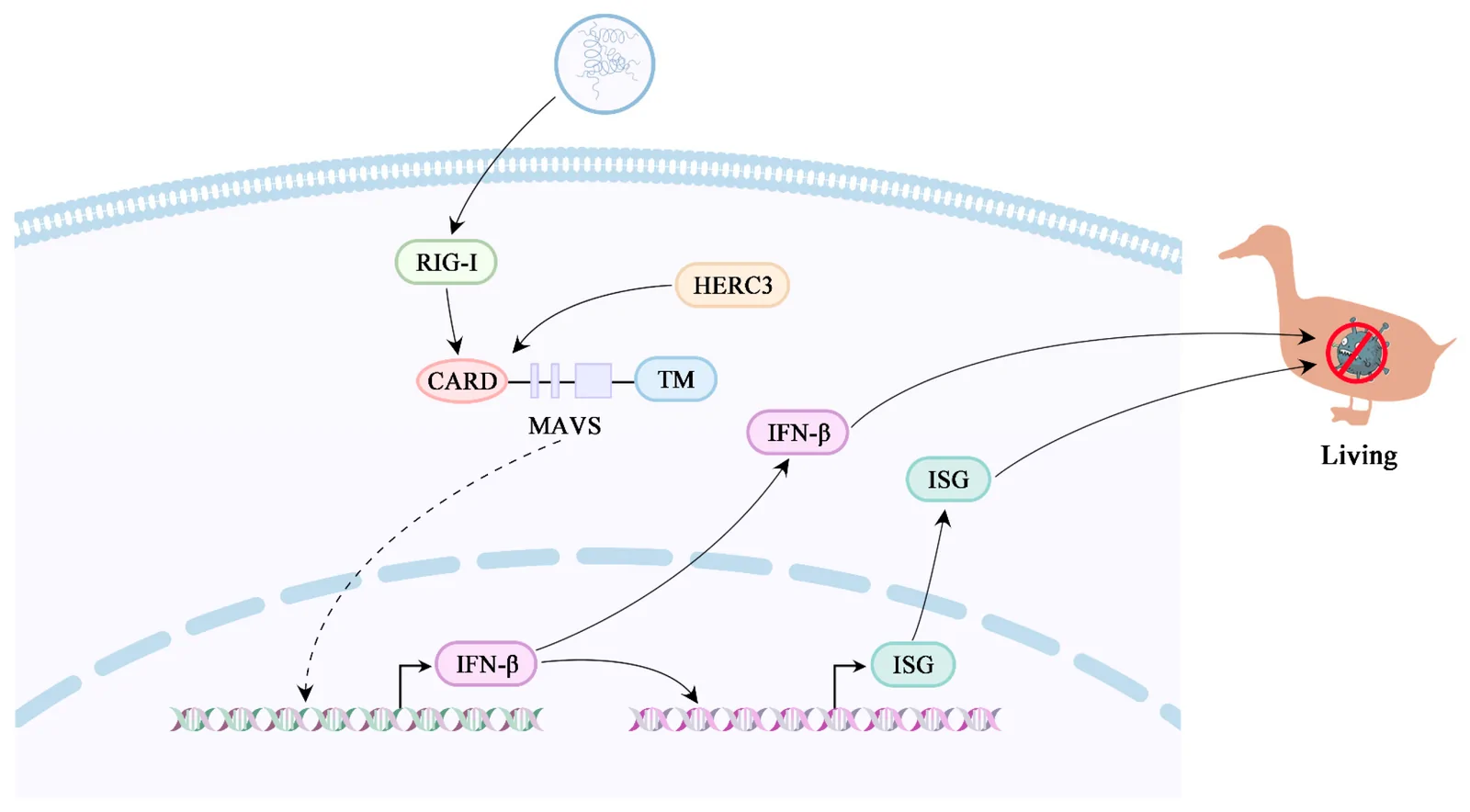
Schematic model illustrating duHERC3-mediated antiviral innate immune regulation via the RLR signaling pathway. Upon viral infection, RIG-I recognizes viral RNA and activates MAVS. duHERC3 physically interacts with the N-terminal domain of duMAVS to promote MAVS transcription, amplifying downstream RLR signaling. This leads to increased expression of IFN-β and the ISG OASL, which collectively mediate antiviral immunity.

To further investigate whether duHERC3 participates in the RLR pathway, we overexpressed and knocked down duHERC3 in DEF^EGFP^ and DEF^RIG-I^ cells. The results showed that duHERC3 overexpression promoted MAVS expression, which in turn upregulated OASL expression and induced IFN-β production. In contrast, knockdown of endogenous duHERC3 significantly reduced MAVS expression and suppressed IFN-β production. HERC3 belongs to the HECT-domain protein family. Several HECT family members, including Smurf1 [24], Smurf2 [25], and AIP4 [26], have been shown to mediate MAVS-regulated antiviral effects. Multiple studies have established that another member, HERC5, plays a central role in mammalian innate immunity by ISGylating viral proteins to disrupt viral replication [27]. However, we did not explore the functional role of HERC3′s ubiquitin ligase activity in the present work, which remains a key focus of our future studies. Through co-immunoprecipitation (Co-IP), we found that duHERC3 interacts with the N-terminal domain of MAVS. It has been reported that multiple proteins positively regulate the RLR pathway through interaction with MAVS. For example, TRIM family members such as TRIM14 [28], TRIM21 [29], TRIM31 [30], and TRIM44 [31] interact with MAVS to promote innate immune responses. OTUD4 interacts with MAVS and removes K48-linked polyubiquitin chains, thereby maintaining MAVS stability and enhancing antiviral signaling [32]. Furthermore, when MAVS was overexpressed together with *duHERC3* knockdown, we observed a significant reduction in IFN-β expression, indicating that the duHERC3-induced increase in IFN-β is MAVS-dependent. Interestingly, overexpression of *duHERC3* together with *MAVS* in the presence of RIG-I led to a marked elevation in IFN-β expression. As RIG-I is the key upstream recognition receptor that senses viral RNA and initiates downstream MAVS-mediated signaling cascades, this synergistic activation effect suggests that duHERC3 does not activate the IFN-β pathway independently, but rather exerts its immunoregulatory function through the canonical RIG-I-MAVS signaling axis. Taken together, our results demonstrate that duHERC3 positively regulates IFN-β production in the RLR signaling pathway through its interaction with the N-terminal domain of MAVS (Figure 6).

## 5. Conclusions

To conclude, duHERC3 is significantly upregulated in tissues of ducks infected with Newcastle disease virus, particularly in immune organs. duHERC3 interacts with MAVS and increases the mRNA levels of interferon-stimulated genes and enhances IFN-β production. Therefore, we have identified duHERC3 as a positive regulator of IFN-β production in the duck RIG-I-like receptor (RLR) signaling pathway. These findings provide deeper insights into the molecular mechanisms by which HERC proteins modulate innate immune responses in ducks and advance our understanding of avian protective immunity.

## Figures and Tables

**Table 1:** Primers used in the study.

Primer Name	Primer Sequence (5′→3′)	Tm/℃	Note
*duHERC3-HA-F* *duHERC3-HA-R*	caagctggctagttaATGCTATGCTGGGGTTACTCGT gctggatatctgcagAGCCAAGCTGAAGCCCTCATAG	68	RT-PCR
*MAVS(aa5-175)-FLAG-F* *MAVS(aa5-175)-FLAG-R*	ttggtaccgagctcgATGGGCTTCGCGGAGGACAAGG aagggccctctagacGTCCAGGTCGGTGCTGGGAACG	68	RT-PCR
*MAVS(aa176-425)-FLAG-F* *MAVS(aa176-425)-FLAG-R*	ttggtaccgagctcgGCCAGGGCCCCTGTGCAGGAAA aagggccctctagacCACTGCTGAGCCCTGGAAAGGG	68	RT-PCR
*MAVS(aa426-614)-FLAG-F* *MAVS(aa426-614)-FLAG-R*	ttggtaccgagctcgGGGGAAAGCTTCGTGAGCCCAT aagggccctctagacGTACACCAGGAAGGCCACGGCG	68	RT-PCR
*duHERC3-F* *duHERC3-R*	TGGGAGGAACTGGAGGAGAG GCCCGTCAGGAACAAGAGAA	60	RT-qPCR
*MAVS-F*	CGAAGCGGACAGGGATGAA	60	RT-qPCR
*MAVS-R*	GGGTTTGGTAGAGGTCGTAGA		
*OASL-F*	TCTTCCTCAGCTGCTTCTCC	60	RT-qPCR
*OASL-R*	ACTTCGATGGACTCGCTGTT		
*duβ-actin-F* *duβ-actin-R*	ATGTCGCCCTGGATTTCG CACAGGACTCCATACCCAAGAA	60	RT-qPCR

**Table 2:** The sequences of siRNAs used in the study.

siRNA	Sequence (5′→3′)
*NC-F* *NC-R* *siduHERC3-1-F* *siduHERC3-1-R*	UUCUCCGAACGUGUCACGUTT ACGUGACACGUUCGGAGAATT GCAACCUCCAGGUCAUCAUTT AUGAUGACCUGGAGGUUGCTT
*siduHERC3-2-F* *siduHERC3-2-R*	GCAUUUGGCUGUGGAACAATT UUGUUCCACAGCCAAAUGCTT
*siduHERC3-3-F* *siduHERC3-3-R*	GCUGGUGGAUCUCUAUAAATT UUUAUAGAGAUCCACCAGCTT
*siduHERC3-4-F* *siduHERC3-4-R*	GCAGAUGCAGGUGGCAAUATT UAUUGCCACCUGCAUCUGCTT

## Data Availability

All data generated or analyzed during this study are included in this published paper.
